# Study to assess the effect of a structured communication approach on quality of life in secure mental health settings (Comquol): study protocol for a pilot cluster randomized trial

**DOI:** 10.1186/1745-6215-14-257

**Published:** 2013-08-16

**Authors:** Douglas MacInnes, Catherine Kinane, Dominic Beer, Janet Parrott, Tom Craig, Sandra Eldridge, Ian Marsh, Joanna Krotofil, Stefan Priebe

**Affiliations:** 1 Canterbury Christ Church University, Canterbury, UK; 2 Kent and Medway NHS and Social Care Partnership Trust, Maidstone, UK; 3 Oxleas NHS Foundation Trust, Dartford, UK; 4 Institute of Psychiatry, King’s College London, London, UK; 5 Queen Mary University of London, London, UK

**Keywords:** Comquol, DIALOG, Forensic, Mental health, Quality of life, Solution-focused brief therapy

## Abstract

**Background:**

Forensic mental health services have largely ignored examining patients’ views on the nature of the services offered to them. A structured communication approach (DIALOG) has been developed with the aim of placing the patient’s perspective on their care at the heart of the discussions between patients and clinicians. The effectiveness of the structured communication approach in community mental health services has been demonstrated, but no trial has taken place in a secure psychiatric setting. This pilot study is evaluating a 6-month intervention combining DIALOG with principles of solution-focused therapy on quality of life in medium-secure settings.

**Methods and design:**

A cluster randomized controlled trial design is being employed to conduct a 36-month pilot study. Participants are recruited from six medium-secure inpatient services, with 48 patients in the intervention group and 48 in the control group. The intervention uses a structured communication approach. It comprises six meetings between patient and nurse held monthly over a 6-month period. During each meeting, patients rate their satisfaction with a range of life and treatment domains with responses displayed on a tablet. The rating is followed by a discussion of how to improve the current situation in those domains identified by the patient. Assessments take place prior to the intervention (baseline), at 6 months (postintervention) and at 12 months (follow-up). The primary outcome is the patient’s self-reported quality of life.

**Discussion:**

This study aims to (1) establish the feasibility of the trial design as the basis for determining the viability of a large full-scale trial, (2) determine the variability of the outcomes of interest (quality of life, levels of satisfaction, disturbance, ward climate and engagement with services), (3) estimate the costs of the intervention and (4) refine the intervention following the outcome of the study based upon the experiences of the nurses and patients. The intervention allows patients to have a greater say in how they are treated and targets care in areas that patients identify as important to them. It is intended to establish systems that support meaningful patient and caregiver involvement and participation.

**Trial registration:**

Current Controlled Trials, ISRCTN34145189

## Background

The UK Department of Health [1] defines *forensic mental health care* as the provision of mental health services for people with mental disorders who are offenders or at risk of becoming offenders. Services are provided in secure, community, NHS and criminal justice settings. The patients include difficult, dangerous and/or extremely vulnerable people whose behaviors present a risk to themselves as well as to others. They can be difficult to engage in assessment, treatment and research, and staff must meet the therapeutic needs of patients while addressing legal, security and public safety issues.

The increasing importance of forensic mental health care can be shown by an increase in medium-secure unit beds in NHS Trusts in England and Wales from 2,500 in 1997 to 3,723 in July 2007 [2]. The average cost of a medium-secure unit bed in London in 2006 was approximately £460 per day [3]. These figures indicate that the annual cost of NHS medium-secure unit places in England is over £500 million. In addition, many prisoners with severe mental illness requiring NHS inpatient care are unable to be transferred, with the principal problem being a shortage of secure psychiatric beds [4].

Developing a valid therapeutic approach has the potential for producing clinical and economic benefits. The *Best Practice Guidance on Specification for Adult Medium-Secure Services*[5] states that the key security factor within a forensic healthcare setting is relational security: the formation of a therapeutic alliance between staff and patients, with individual patient care at the center of relational security. It also recommends developing patient involvement in all aspects of the service. This publication followed earlier guidance that the interests of patients should lead service developments and that treatment should be guided by their aspirations and experiences [6]. However, a review of forensic mental health services noted a lack of a patient perspective and involvement in the service [7]. The report suggested future work should seek to build mechanisms and services that involve patients and respond to their views. This is supported by research findings that significantly better clinical outcomes are reported, with reductions in unmet need, lower levels of psychopathology, higher global functioning, lower social disability, higher quality of life and better satisfaction with services occurring when an agreed clinician–patient intervention strategy is in place [8,9]. However, there is a lack of research in forensic settings concerning therapeutic relationships, and there has been no published research regarding examining relational security in secure settings. It has been proposed that quality-of-life assessments may represent the only way of measuring the totality of detained forensic patients’ experiences in secure environments to guide the development and improvement of patient care [10].

Research in primary care settings indicates a patient-centered approach, including active participation of patients in the treatment process, is associated with better quality of life, increased adherence to treatment regimens and reduced misunderstandings between clinicians and patients [11,12]. A positive relationship with the primary worker is consistently found to predict a better outcome in relation to symptomatology, time in hospital and quality of life [13]. Priebe and colleagues [14] have developed an intervention using a structured communication approach that places the patients’ perspective of their care at the heart of the discussions between patients and clinicians. The intervention consists of two elements: a computer-mediated approach (DIALOG) used in conjunction with nondirective counseling based on the principles of solution-focused brief therapy (SFBT). This has been found to be an effective, practical method of developing patients’ involvement in their treatment. In DIALOG, the patient completes a simple assessment in the meeting with the clinician, recording the degree of satisfaction with a range of life and treatment domains. In an updated version of the intervention, the rating for each domain is entered onto a tablet. A tablet is a mobile computer that is operated primarily by touching the screen, with iPads (Apple, Cupertino, CA, USA) being used in this study. Patients are also asked if they require additional help to improve their current situation in a given domain. Areas where help is requested are then marked, and requests for additional help remain visible. Upon completing the ratings, the patient and the clinician can see an overview of all domains and may compare current scores for each of the domains. All of the previous ratings can be displayed graphically on the tablet screen and used to monitor developments over time. The ratings are intended to inform the conversation between the patient and the clinician, with some or all of the ratings discussed in detail. In a trial in six European countries, the intervention group had significantly higher quality-of-life scores, more satisfaction with treatment and fewer unmet needs compared to the control group. A major strength of the study is that the intervention was tested in routine clinical conditions.

The underlying rationale of this approach is that providing patients and nurses with this information will lead to explicit negotiations about what each individual patient wants and what the nurse can help do about it. The hypothesis presented is that this focus on the individual concerns of the patient will in turn lead to an improvement in subsequent care and the patient’s quality of life.

In a recent paper, it was found that the psychometric qualities of subjective quality-of-life scores generated in DIALOG are strong. This indicates that DIALOG ratings can also be used to evaluate quality-of-life scores, adding to the value of the approach [15]. DIALOG has not been tested in a forensic environment. It is proposed that using a structured patient–clinician communication approach within a forensic mental health setting would improve patients’ quality of life, levels of satisfaction and engagement with services and would reduce disturbance. There is, however, a need to pilot the intervention in this setting to establish the viability of a large full-scale trial, determine the variability of the outcomes of interest, estimate the costs of the intervention and refine the intervention following the outcome of the study based upon the experiences of the nurses and patients.

## Methods and design

### Design

**Figure 1:**
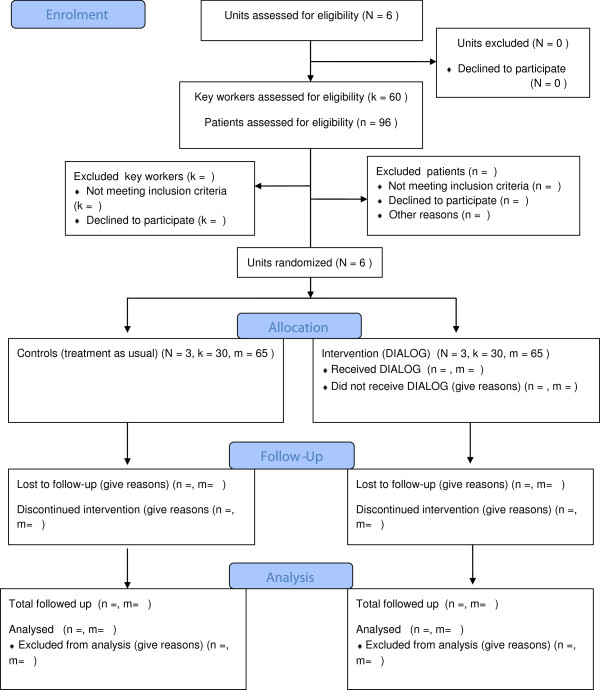
Comquol flow diagram.

A pragmatic cluster randomized trial has been designed, avoiding any potential contamination between the intervention and control groups in clinical practice. Six medium-secure units will be randomized. Far fewer women than men are resident in secure units. To enable the study to examine the intervention with both men and women in the forensic mental health service, the units are stratified. The first stratum includes four medium-secure units with two male wards in each unit participating in the study. The second stratum consists of two medium-secure units, with one male ward and one female ward in each unit participating in the study. Within both groups, there is a balanced design, resulting in the same number of units in each of the intervention and control groups (Figure 1). A randomization schedule has been drawn up by a statistician independent of the study. The study will include a population of inpatients in the six forensic medium-secure wards. A 6-month intervention approach is being used based on the work of Priebe *et al*. [13,14].

### Ethical approval

Ethical approval was obtained from the London Surrey Borders Research Ethics Committee in July 2011 (reference number 11/LO/0104).

### Participants

The participants are registered mental health nurses and inpatients at six medium-secure units in southern England and London. Nurses will initially be approached in two wards in each of the participating units (12 wards overall). The inclusion criterion for the clinicians will be that they are registered mental health nurses working with inpatients within those wards. Each patient residing in the participating wards is eligible to participate as long as the following inclusion criteria are met: They must have a history of least 3 months of current inpatient treatment in the service and are capable of giving informed consent. There is no time frame for the recruitment of participants. Recruitment will take place until a sufficient number of nurses and patients have agreed to participate. If there are insufficient numbers of participants from the two identified wards, discussions will be held with the clinicians and management teams of the unit to consider whether other wards are able to be included. Informed consent from both nurses and patients is obtained before they are included in the study. To avoid bias, the allocation of a unit into either the intervention or control arm of the study is undertaken only following the identification and recruitment of a sufficient number of nurses and patients from each unit [16]. The researchers are therefore blinded to allocation status at the point of nurse and patient entry into the study. The allocation is performed by the randomization service of the registered Pragmatic Clinical Trials Unit (PCTU) at Barts and The London School of Medicine and Dentistry.

Pilot studies are limited in terms of the number of participants involved, and it is acknowledged there may be some nurses and patients who leave the study during the 12-month period from baseline assessment to 6-month postintervention assessments. This may occur for various reasons, such as a nurse moving to a different job or a patient moving to another nonsecure unit as treatment progresses. Attempts will be made to follow up all patients who drop out of the study, unless the patient requests to withdraw from the study.

Ten nurses from each ward in the intervention units will be trained in the structured communication approach to allow for some dropouts. The trial will recruit 96 patient participants (48 each in intervention and control arms) to allow for some dropouts. The findings of this study will be used to estimate variability in outcomes for a later, larger study. As this is a pilot study, a formal sample size calculation is inappropriate.

Many complex interventions require a long-term commitment and often necessitate significant effort from participants. Therefore, the process of recruitment, participation and follow-up will be monitored to ensure that the intervention is acceptable and meaningful to patients and clinicians. The monitoring will take place through structured monthly meetings to be held between a research team member and those involved in the intervention and an in-depth exploration of the patients’ experiences through focus group discussions. The loss of participants over the course of a trial will also be carefully monitored with the offer of follow-up interviews to all participants who either withdraw or drop out of the study, and reasons for leaving the study will be explored with those participants. A nominal sum of £35 will be given to each patient participant upon completion of each set of assessments.

### Interventions

Participants allocated to the intervention group will receive the DIALOG approach combined with counseling guided by SFBT. The DIALOG approach involves monthly meetings between the patient and nurse for a period of 6 months arranged as part of routine care. The intervention consists of two elements: a computer-mediated approach in conjunction with nondirective counseling, which has been found to be an effective practical method of developing patients’ involvement in their treatment. DIALOG is used by nurses nominated to facilitate structured communication sessions to enable individualized therapeutic discussions. During the meeting, the patients complete a simple rating list, recording their degree of satisfaction in a range of life and treatment domains. The 11 domains are mental health, physical health, accommodation, job situation, leisure activities, friendships, relationship with family and/or partner, personal safety, practical help, meetings and medications. Each domain is rated on a scale from 1 to 7 (from “couldn’t be worse” to “couldn’t be better”) and is followed by a question about whether the patient wants any additional or different help in the given domain. If the patient answers yes, the type of the requested additional or different support is discussed and recorded. The 11 domains are presented in a fixed order, and an explicit response is required for each item before proceeding to the next item. Participants’ answers to all questions are entered directly onto a tablet using specifically developed software. The tablet allows patients and nurses to view screen displays detailing the current rating of a domain as well as the rating from any previous month. The procedure is designed to ensure that patients’ views on their situation and needs for care are the central point of treatment discussions and that their views on what kind of help would improve their current situation is explicit.

The counseling approach offered is the solution-focused approach. It is a structured conversational approach that promotes movement toward positive change in individuals, families and systems. It is based on SFBT. The approach is characterized by a focus on the future––more specifically, exploring what will be different when things are better.

A 3-day training program will be delivered to all nurses in the intervention group in two areas to help ensure that the DIALOG approach is consistently administered. Each nurse in the intervention group will be individually trained to use the software by a researcher and will be provided with written instructions. The nurses are instructed on how the ratings should be used to facilitate a dialogue with the patients, particularly when there are changes since the previous rating, when there is explicit dissatisfaction with life domains or treatment aspects or when the patient wants additional or different support. Nurses facilitating structured communication sessions also receive a training program in SFBT. The training will be delivered by an experienced SFBT therapist who runs a master’s course in SFBT and is a founding member of the United Kingdom Association for Solution Focused Practice. Each nurse will also receive a practical handbook explaining how to conduct the solution-focused approach to help ensure that a similar approach is used in all sessions.

Patients in the control arm will meet nurses with the same frequency as those in the intervention group. The meeting will be used to plan and evaluate care as well as to discuss any specific difficulties, but without using the formalized DIALOG approach.

### Monitoring the intervention

The nature and variation of the approaches used by different nurses will be recorded and evaluated. To enable a clear description of the ways in which the sessions are conducted and to try to ensure a consistent approach by the nurses, the main topics of each session are recorded by the nurse facilitating these sessions on a record sheet at the end of each session. Furthermore, each nurse and patient are asked to consider whether up to two sessions of the six-session intervention can be audiotaped. In addition, monthly meetings are held between a researcher and each nurse to examine the intervention and monitor the needs of the nurse. This guides the acceptable limits to which practitioners can individualize interventions and help ensure that individual styles and evolution in treatment do not render results from different groups of participants incomparable or excessively reduce the effectiveness of the intervention.

### Assessment

#### Feasibility

To establish the feasibility of a full-scale cluster randomized trial, we will calculate the estimated treatment effects and corresponding confidence intervals for all outcomes measured at 6 and 12 months. These outcomes are detailed in the following section. We will first calculate a mean value of the outcome for each unit and then calculate the treatment effects (and corresponding confidence intervals) as the mean difference of these means in the intervention and control groups. This is an acceptable method of analyzing data from cluster randomized trials when the number of clusters is small. Given that this is a pilot study, we expect confidence intervals to be wide, and hypothesis testing will be inappropriate. We will instead use the confidence limits of each treatment effect and knowledge of the clinically important difference for each outcome (where this is available) to determine whether clinically important differences are ruled out by these confidence limits. If they are not, then this will indicate that it is worth pursuing a full-scale trial. However, where confidence intervals indicate an unambiguously clinically important effect, we will test the statistical significance using *t*-tests of data aggregated to the cluster level. The aggregation to cluster level will be carried out as already described. We will also consider the recruitment and dropout rates to assess the feasibility of conducting a full-scale trial with similar rates. For the outcomes measured monthly, we will plot data graphically to look at patterns and whether there are any obvious trends. This will facilitate the construction of possible suitable outcome measures for the full-scale trial from these data. In addition, for each outcome measure at each time point, we will examine completion rates, both whether the outcome measure has been completed at all and the extent of completion. This will indicate the feasibility of using the various outcome measures in a full-scale trial and also help to determine the likely size of the trial if we allow for dropouts in a sample size calculation. In addition, the number of participants recruited from each ward, the number of staff and patients who refuse to take part, as well as the number of drop outs will also be recorded. We will be collecting and summarizing data on the attendance of nurses at training events and the timing of the expected monthly sessions between nurses and clients and between nurses and a researcher, to ascertain the fidelity of the intervention. Finally, demographic data collected on each participant at the start of the study will also be used to describe the participants in this study. The research team will reflect on the representativeness of the demographics in terms of the general population of patients that could potentially be use the intervention.

#### Outcomes

##### Primary outcome measures

The primary outcome is quality of life. This will be assessed by administering the Manchester Short Assessment of Quality of Life (MANSA) [17], which has 16 questions with responses recorded on a seven-point Likert scale. The questions will be given to the participants prior to them being contacted by a researcher blinded to the allocation status of the participants. The researcher will interview the participants by telephone, asking the MANSA questions and noting their responses.

The primary end point will be measured at three time points. To ensure that results are not affected by a secular trend, each intervention group unit will be paired with a control group unit. The assessments for each paired intervention–control group unit will be carried out within 1 month of each other at each time point. Time point 1 is the baseline assessment of patients. For the intervention group, this will be prior to their first structured communication session, and for the control group, it will be at the same time as noted above. Time point 2 occurs within the 2 weeks following the intervention, which is the last DIALOG approach meeting (after 6 months). Time point 3 is at 6 months postintervention (12 months after time point 1).

##### Secondary outcome measures

The secondary outcome measures are disturbance, therapeutic relationships, social climate of ward, recovery measure, nurses’ stress and satisfaction checklist. Each is discussed in turn in the following paragraphs.

*Disturbance*. Any disturbed behavior involving the patients will be taken from the ward untoward incident forms and patient progress notes on a monthly basis from 3 months prior to the assessment until the 6-month postintervention follow-up (15 time points). The behaviors recorded are the number (and hours) of seclusions, the number of incidents where the patient was physically restrained, the number of suicide attempts, the number of attempts at self-harm, the number of violent acts toward others, the number of violent acts toward inanimate objects, the number of attempted abscondings or escapes and the number of actual abscondings or escapes.

*Therapeutic relationships*. The perceived value of the therapeutic relationship will be assessed using the Helping Alliances Scales [18].

*Social climate of the ward*. The therapeutic milieu of the ward environment will be measured by using a 17-item questionnaire for assessing the social climate of forensic psychiatric wards: the Essen Climate Evaluation Schema (EssenCES) [19].

*Patient satisfaction*. Patient satisfaction will be assessed using the 60-item Forensic Satisfaction Scale [20]. This rates service-user satisfaction in seven domains (staff interaction, rehabilitation, communication, milieu, finance, safety and overall care).

*Recovery measure*. The Questionnaire about the Process of Recovery (QPR) will be used to measure recovery [21]. The QPR is a 22-item scale that asks about aspects of recovery that are meaningful to patients.

*Nurses’ stress*. Nurses’ stress will be assessed using the 22-item Maslach Burnout Inventory at the three identified time points [22].

*Satisfaction checklist*. Only the intervention group will complete the satisfaction checklist to generate more information for the evaluation of the intervention. Patients will complete the checklist during each monthly structured communication session. The checklist rates their level of satisfaction with 11 life domains (mental health, physical health, accommodation, job situation, leisure activities, friendships, relationship with family and/or partner, personal safety, practical help, meetings and medications). Each domain is rated on a scale of 1 to 7 (from “couldn’t be worse” to “couldn’t be better”).

##### Outcome assessments

To assess the likely size required for a full-scale trial, we need some estimate of the variability of the primary outcome and, in particular for a cluster randomized trial, an estimate of the intracluster correlation coefficient (ICC). Relying on pilot studies or even on single previous trials for reliable estimates of the ICC is problematic because sampling errors are very large. Nevertheless, the randomization units in this study are unusual, and ICCs from a number of other similar studies (the safest way of calculating ICCs) will not be available. Because of this circumstance, we will calculate an ICC from this study using standard analysis of variance techniques, although we will also attempt to triangulate this with other information available if possible.

### Economic costs

Using economic analysis, we will examine the feasibility of a full-scale cost-effectiveness evaluation of the intervention compared to standard treatment from the perspectives of health, social and criminal justice services. A key task of the economic evaluation will be estimating the cost of the nurses’ training in the DIALOG approach plus SFBT-based counseling. We will measure the time taken for training and ongoing supervision and estimate the cost of using the methodological approach set out by Netten and colleagues [23]. The training costs will be considered in both the short term and the long term, assuming that the training will continue to accrue benefits into the future.

In addition to the costs of the intervention, the services used by participants will be studied. The number of contacts with nurses will be collected from files. The Secure Facilities Service Use Schedule (SF-SUS) [24] will be used to collect data on all other service use over the 12 months of follow-up. The SF-SUS is completed with information taken from patient files and records on accommodation, health and social care contacts, contacts with criminal justice professionals and daily activities, including education and therapeutic and physical activities, both within the secure facility and in external services. The schedule could thus also be used to collect patient information for those in the study who may be discharged at follow-up. The SF-SUS has been piloted, tested and successfully applied in previous research in secure facilities [25,26].

The cost per night in each of the units will be calculated using local data wherever possible. Costs accrued outside the facility will be calculated using existing unit cost estimates [23,27]. Resource use differences between the intervention and control groups will not be compared statistically to avoid problems associated with multiple testing. Instead, resource use patterns will be described without statistical comparisons. Total costs will be calculated as the costs of time in the units, any external costs and, for the intervention group, the cost of training the nurses in the DIALOG approach plus SFBT.

Costs and outcomes will be combined in a cost–consequences analysis whereby costs are reported alongside a range of outputs [27]. The cost-effectiveness of the intervention compared to standard care will be assessed using primary (quality of life) and secondary (disturbance) effectiveness outcomes. Incremental cost-effectiveness ratios (ICERs) will be estimated as a cost per 1-point improvement in the patient’s MANSA satisfaction scale score for the primary outcome and as a cost per incident avoided (for example, violent act, suicide attempt, escape) for secondary outcomes. The small number of clusters and participants in this pilot study does not permit an assessment of uncertainty around the ICER point estimates using probabilistic methods, nor does it allow for adjustments for the cluster randomized design. Nevertheless, we will identify the major sources of uncertainty and estimate the impact of this uncertainty on ICERs in deterministic sensitivity analyses.

### Participants’ experiences

#### Evaluation of patients’ experiences

Following the final session, three focus groups will be convened (one in each intervention unit) to draw out respondents’ attitudes, feelings, beliefs and experiences regarding the intervention [28]. Each group will contain between four and eight patients and last 60 to 90 min. If more than eight patients wish to take part, a further group will be formed. The focus group discussions will be audiotaped and transcribed.

There is strong support for the view that individuals who are similar to the target audience for the project are involved in this process, and one patient member of the research team is involved in developing the interview schedule and moderating the groups [29]. Training in running focus groups is given by one of the research team members (IM) to ensure competence in focus group techniques [29]. This approach has been undertaken successfully in a previous project involving members of the project team [30].

#### Interviews with nurses facilitating structured communication sessions

Monthly interviews will be held with the nurses from their first meeting to their final session. The research team member records the following information at each monthly meeting: types of concerns and problems identified, types of approach used to find solutions to the problems and acceptability of the approach from the nurse’s perspective.

#### Follow-up interviews for participants who withdraw or drop out of the study

Each patient and each nurse who withdraws or drops out of the study will be offered an individual interview to discuss their reasons for withdrawing or dropping out. The main reasons for leaving and any suggestions for improving the conduct of the study will be recorded.

#### Data analysis

The data from the three meetings will be analyzed using thematic analysis. This focuses on describing identifiable themes and patterns of living and/or behavior [31]. In the analysis, we will examine the nurses’ and patients’ views of the interventions and outcome measures used in the study. The findings will be fed back to respondents for validation or revision of the interpretations.

## Discussion

The aim of this study is to undertake a pilot trial to examine the proposed methodology of an intervention study based upon the structured communication approach developed by Priebe *et al*. [13]. The value of a pilot study lies in the understanding it generates concerning the study procedures in providing a thorough evaluation of the acceptance and feasibility of the proposed approach.

The following are the specific objectives of the study: (1) establish the feasibility of the trial design as the basis for determining the viability of a large full-scale trial (estimated treatment effect, study procedures, outcome measures, estimates of recruitment for a main trial and follow-up of participants); (2) determine the variability of the outcomes of interest (quality of life, levels of satisfaction, disturbance, ward climate and engagement with services); (3) estimate the costs of the intervention; and (4) refine the intervention following the outcome of the study based upon the experiences of the nurses and patients.

An important element of care in forensic mental health services is the development of good therapeutic relationships with patients and the subsequent impact on their recovery and relational security. The findings from this study will contribute to these aspects of service delivery in a number of ways. The findings will inform the delivery of care in NHS settings by providing access to a structured and clearly detailed intervention approach that can be incorporated into practice standards in forensic mental health care settings. If the pilot trial and subsequent full trial show that the personalized DIALOG intervention is effective, the findings may have important implications for the treatment approaches used in forensic mental health care settings worldwide. If the implications of this study are positive, they will also provide guidance regarding which interventions may be most suitable for different quality-of-life domains.

One of the strengths of the proposed research is patient involvement in the planning and running of the study. This has been recognized by the Mental Health Research Network (MHRN) with the MHRN User Involvement Prize awarded to the Comquol study in 2012. The current proposal was developed following a collaborative research project involving one member of the research team (DM) and three patients. This resulted in the development of the Forensic Satisfaction Scale [20], one of the secondary outcome measures being used in this study. The experience gained during this collaborative work has guided the procedures being used to ensure patient input into the project [32]. The proposal was presented and reviewed at a patient forum at a medium-secure unit. It was discussed with the patients and caregiver members of the MHRN clinical research group Service Users’ Experience of Secure Settings (SUCESS). It was also subject to peer review as part of the requirements of the funding process of the Research for Patient Benefit program.

Two patients were involved in formally reviewing the proposal, and their comments were used to amend the proposal. One patient also agreed to participate as a member of the research team and meets monthly with members of the project management team to review the conduct of the research. Patients will also be involved in the analysis of the data and in the dissemination of the findings. The patient member of the research team will also lead the part of the project examining the experiences of interventions from the patient participants’ perspective. This will involve training in focus group techniques and thematic analysis that will be provided by a member of the research team.

### Limitations

Although the present study meets many requirements for a high-quality clinical trial, there are some potential limitations of its design which need to be taken into consideration. One limitation is the difficulty in maintaining consistency in relation to allocation of the nurse facilitating the structured communication sessions for a given patient. As patients progress through the care pathway and move between wards within the units, they are retained in the study sample and continue receiving the intervention based on the structured communication approach; however, the nurse facilitating these sessions would be one of the nurses based in the new ward.

Another limitation is related to the possible selection bias. The use of patient volunteers may lead to selective participation from subjects with particular characteristics, such as higher level of motivation to engage in therapeutic relations or more generally in any type of activity. To minimize selection bias, a monetary incentive has been introduced to encourage participation of patients who are less likely to engage in ward-based activities. Patients allocated to the intervention and control groups are offered the same financial incentive.

## Trial status

The trial was actively recruiting participants at the time that the protocol was submitted for publication.

## Abbreviations

Comquol: Structured communication approach on quality of life; EssenCES: Essen climate evaluation schema; ICC: Intracluster correlation coefficient; MANSA: Manchester short assessment of quality of life; MHRN: Mental Health Research Network; PCTU: Pragmatic clinical trials unit; QPR: Questionnaire about the process of recovery; SFBT: Solution-focused brief therapy; SF-SUS: Secure facilities service use schedule; SUCESS: Service users’ experience of secure settings.

## Competing interests

The authors declare that they have no competing interests.

## Authors’ contributions

DM is the chief investigator of the study and coordinated and wrote the application to the National Institute for Health Research. DB helped develop the original proposal. DM, CK and JP share day-to-day management of the project. TC is the project supervisor. SP developed the intervention approach and oversees the implementation of intervention. SE oversees the statistical and economic analysis. IM is collecting and analyzing qualitative data. JK is the project research assistant who has recruited participants and collected the study data. All authors contributed substantially to the study design, development of the intervention, protocol writing and drafting of the manuscript. All authors read and approved the final manuscript.
